# Modeling social cognition in alcohol use disorder: lessons from schizophrenia

**DOI:** 10.1007/s00213-024-06601-0

**Published:** 2024-05-18

**Authors:** Irene Perini, Arthur Pabst, Diana Martinez, Pierre Maurage, Markus Heilig

**Affiliations:** 1 Center for Social and Affective Neuroscience, Department of Biomedical and Clinical Sciences, Linköping University, Linköping, Sweden; 2 Center for Medical Image Science and Visualization, Linköping, Sweden; 3 Louvain Experimental Psychopathology research group (LEP), Psychological Sciences Research Institute, UCLouvain, Place C. Mercier 10, Louvain-la-Neuve, B-1348 Belgium; 4 Columbia University, New York State Psychiatric Institute, New York, NY 10032 USA

**Keywords:** Alcohol use disorder, Social cognition, Social behavior, Facial expression, Personality traits, Translational research

## Abstract

A better understanding of social deficits in alcohol use disorder (AUD) has the potential to improve our understanding of the disorder. Clinical research shows that AUD is associated with interpersonal problems and the loss of a social network which impedes response to treatment. Translational research between animal models and clinical research may benefit from a discussion of the models and methods that currently guide research into social cognition in AUD. We propose that research in AUD should harness recent technological developments to improve ecological validity while maintaining experimental control. Novel methods allow us to parse naturalistic social cognition into tangible components, and to investigate previously neglected aspects of social cognition. Furthermore, to incorporate social cognition as a defining element of AUD, it is critical to clarify the timing of these social disturbances. Currently, there is limited evidence to distinguish factors that influence social cognition as a consequence of AUD, and those that precede the onset of the disorder. Both increasing the focus on operationalization of social cognition into objective components and adopting a perspective that spans the clinical spectrum will improve our understanding in humans, but also possibly increase methodological consistency and translational dialogue across species. This commentary underscores current challenges and perspectives in this area of research.

## Defining AUD through social cognition in humans

Addiction research, including that on AUD, has largely focused on substance use as the outcome measure in clinical trials. Use is quantifiable, and validated tools allow for its reliable assessments, through self-reports such as the Time-Line Follow Back (Sobell and Sobell 1992), or objective biomarkers such as phosphatidylethanol and ethyl glucuronide (Wurst et al. 2015). Both pharmacological and behavioral AUD treatments with support for efficacy to reduce use have been developed (Witkiewitz et al. 2019). Among these, available medications include naltrexone, acamprosate and others, while Motivational Enhancement, Relapse Prevention, Community Reinforcement Approach and Contingency management are examples of behavioral interventions with support for efficacy. However, effect sizes of these treatments are insufficient. Furthermore, although research on molecular, neural and psychological mechanisms of alcohol-related behaviors has grown, no mechanistically novel treatments have been approved by the Food and Drug Administration (FDA) or the European Medicines Agency (EMA) in two decades (Heilig et al. 2019). The situation is similar for psychological treatments, where available treatments are based on long-established cognitive-behavioral principles. Collectively, large unmet needs for AUD treatments remain, and could potentially be addressed through approaches that go beyond those used to date. An opportunity for achieving this objective may be by improving the understanding of social processes that contribute to initiation and maintenance of AUD, a condition characterized by a vicious circle of progressive social marginalization and impairment (Heilig et al. 2016).

People with AUD face a range of social difficulties that negatively impact their relationships, employment, and overall quality of life (e.g. (Levola et al. 2014). These interpersonal problems can also impair recovery, as shown by the inverse relationship between social support and relapse (Havassy et al. 1991) and research showing that negative emotions and interpersonal conflict constitute stronger determinants of relapse than cognitive functions (e.g. (Sliedrecht et al. 2019). Furthermore, the pervasive and chronic social functioning impairments in AUD may not improve with decreased alcohol use, compromising treatment efficacy. Thus, understanding social cognition associated with AUD may be essential for improving treatment, reducing relapse rates, and more generally, decreasing the substantial social stigma and social exclusion related to AUD (Kilian et al. 2021).

**Fig. 1:**
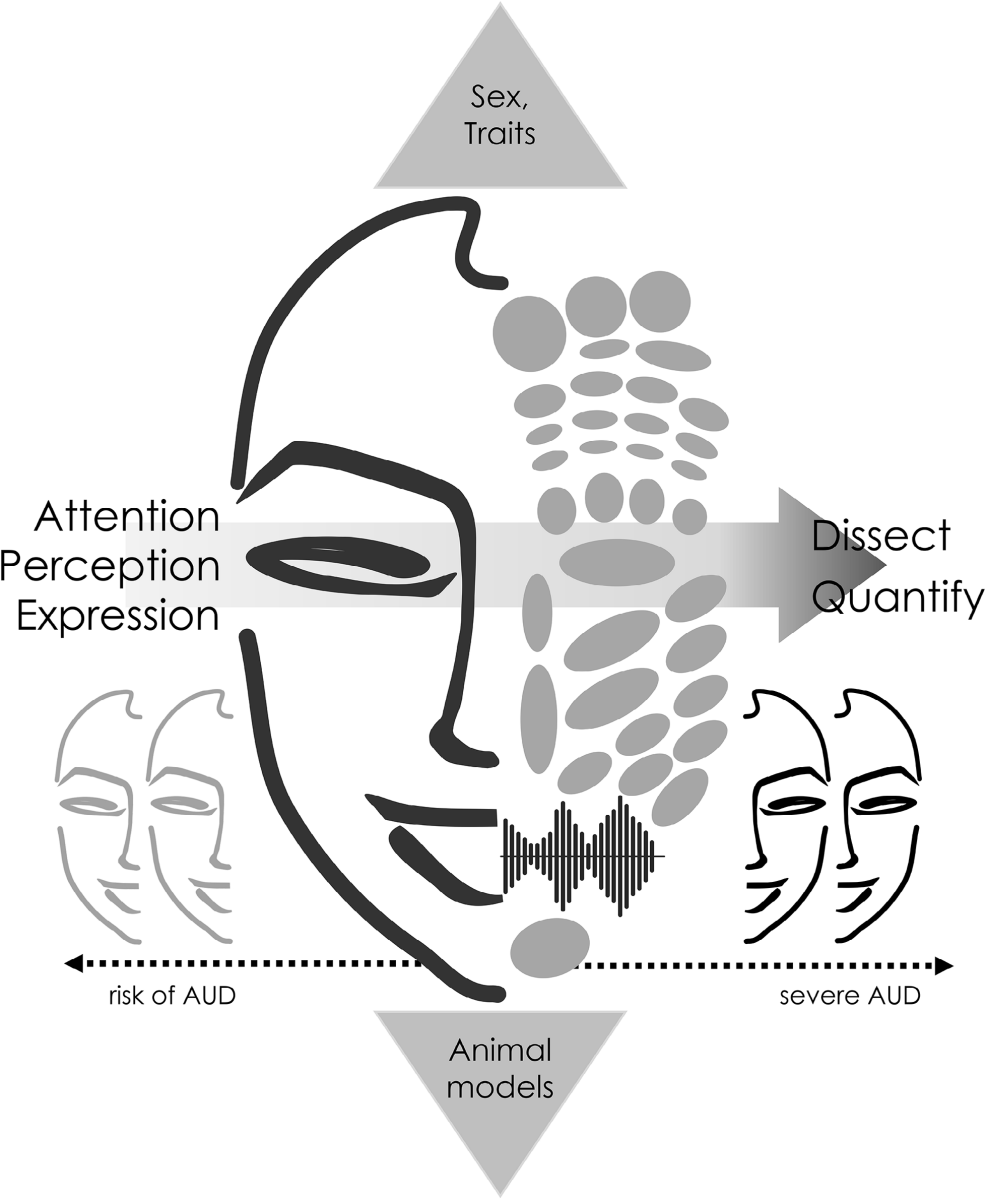
Individuals with AUD often experience interpersonal difficulties. This commentary advocates for the incorporation of social cognition as a defining element of AUD. It proposes a reflection on two overarching topics. The first emphasises the importance of dissecting and quantifying social cognition into tangible components. The second underscores the significance of distinguishing factors that influence social cognition as a consequence of AUD from those that precede the onset of the disorder. To this end, we first emphasize the importance of profiling social cognition across the severity continuum in humans and highlight the utility of using animal models, which offer greater feasibility in addressing large timespans. Finally, we emphasize addressing stable factors like sex and personality traits to understand their impact on social cognition and their potential role in predisposing individuals with AUD to social challenges

This commentary aims to underscore the significance of social cognition as a defining factor in AUD by addressing pertinent topics in its investigation. After a critical review of earlier findings and methods classically used to assess social cognition in AUD, the first section suggests employing assessments that achieve a balance between ecological validity and standardization. Emphasis is placed on methods that have been employed in schizophrenia research, where automated extraction of indexes of social cognition, in the form of facial units and vocalizations, proved to be valuable in characterizing clinical symptomatology and diagnosis. The second section will focus on the significance of profiling social cognition across the severity continuum to differentiate between disruptions in social cognition that directly result from problematic drug use from those operating prior to it. Social exclusion is a key part of the cycle of alcohol use, craving and relapse (Brownell et al. 1986), yet it often manifests as a phenomenon in more severe cases. Thus, it is important to understand the factors *leading* to social exclusion and to map social cognition at different stages of the clinical continuum, including in people at risk of AUD. This can be achieved with both cross-sectional and longitudinal studies. The latter are cumbersome and time consuming in humans but are more feasible in animal models. Therefore, while being mindful of species differences in social behaviors, animal models can provide a unique support in the characterization of core social functioning and severity at different timepoints. These topics will be discussed in the second section. In assessing the clinical severity continuum, it is as important to gather information on relatively stable factors, such as personality traits and sex, to disentangle impairments of social cognition arising from problematic alcohol use from those that are pre-existing. To this end, the last section will highlight the significance of mapping personality traits, with relevance on impulsivity and antisocial tendencies due to their prevalence in individuals with AUD. Monitoring personality traits in social cognition in AUD would enhance our understanding of their impact on social cognition and whether they predispose individuals with AUD to face social challenges. See Figure 1 for a summary of the topics of this commentary. 

## Dissecting social cognition

Socialization is the result of a high-order multidimensional, integrative cognitive processes (Perini et al. 2022) that change over-time. Social cognition refers to the mental processes involved in perceiving, interpreting, and understanding information about oneself and others while interacting with others. It encompasses a range of cognitive abilities, including recognizing emotions, understanding social cues, making inferences about others’ thoughts and intentions, as well as *acting* appropriately depending on the situation. Given its multidimensional nature, characterizing social cognition in AUD should encompass an evaluation of its subcomponents, beyond a global “social cognition” score. To this end, a scientific consensus on key measures for the characterization of social cognition in AUD should be reached, as done for example in schizophrenia research (Green et al. 2008). One critical challenge in pursuing this goal is also the identification and implementation of tools that can guarantee a good balance between experimental control and ecology. Technical advances in the processing of digital data can allow for more ecologically valid, yet standardized and objective assessments of the perception and interpretation of social stimuli, and potentially even allow interactive features of socialization to be studied. Such assessments, of which a few are highlighted below, can uncover latent processes that would shed light on the nature of impaired social function in AUD.

One frequently investigated domain of social cognition in AUD is emotion recognition. These tasks assess the ability to decode and recognize facial expressions, which are presented as a picture of a person’s face while asking participants to choose the word that describes the emotion. Two recent meta-analyses on emotion recognition show a decreased ability to recognize facial expressions in people with AUD, including recently detoxified individuals, compared to healthy controls (Bora and Zorlu 2017; Castellano et al. 2015). Additionally, some studies have shown that individuals with AUD have lower accuracy in identifying negative emotions such as disgust and anger, while their ability to recognize positive emotions appears to be preserved (Bora and Zorlu 2017; Castellano et al. 2015). While potential sex effects were investigated in these meta-analyses, no significant findings were reported. This is likely due to insufficient sample sizes that hindered formal statistical comparisons. A systematic investigation of sex effects in emotion recognition is needed.

Overall, although these meta-analytical findings suggest that AUD is associated with impaired emotion recognition, there are some limitations, as described recently (Pabst et al. 2022; Pabst & Maurage, 2023). For instance, emotion recognition tasks may include non-social confounders that are not fully controlled for. For example, performance on typical emotion recognition tasks may be affected by categorical decision-making or emotional word processing difficulties. In addition, perhaps the biggest limitation of classic emotion recognition paradigms using static facial expressions is their relatively low ecological validity. They often rely on static and decontextualized stimuli that don’t fully capture the dynamic and integrative nature of socialization (Perini et al. 2022). Overall, while classic emotion recognition tasks provided initial evidence on emotion recognition processing in AUD, their limitations underscore the importance of implementing more ecologically valid tools.

Video-based depictions of social situations provide greater context when gauging emotion recognition and theory of mind (i.e. the ability to understand other’s mental beliefs, states and intentions), the latter being another domain commonly assessed in AUD. The Movie for the Assessment of Social Cognition (MASC) (Dziobek et al. 2006) allows the assessment of subtle difficulties in extracting the social information needed to infer socially common interpretations of other people’s states. Initially designed to assess social cognition in individuals with Asperger’s syndrome, it consists of a 15 min movie of a dinner party with four characters. Each character has defining traits and their social interactions represent different emotional and cognitive states in the characters. These emotions are conveyed through a combination of verbal means, encompassing both literal and figurative language, as well as non-verbal cues, spanning from facial expressions to bodily movements and gestures. The ability to infer emotions and intentions are assessed via questions presented during the video, to assess participants’ ability to recognize different degrees of inferential complexity, including deception, sarcasm, irony and first and second order false belief. Another validated audiovisual instrument in the assessment of social inference, is the “The Awareness of Social Inference Test” (TASIT (McDonald et al. 2003). Here, brief videos showing straightforward social scenarios investigate the ability to recognize basic emotions, and the ability to differentiate between literal from counterfactual social exchanges, such as sarcasm and lies. The ability to detect sarcasm and lies requires integrative cognitive efforts in which auditory and contextual information is integrated with visual cues. Although the TASIT was initially developed for assessing social inference difficulties in individuals with traumatic brain injury (TBI) (McDonald et al. 2003) it has also been used to assess social cognition in individuals with psychiatric diagnosis. For example, in individuals with schizophrenia, TASIT performance has been used to define clusters based on level of social impairment (Rocca et al. 2016) and to assess the association between improvements in clinical and social inference domains (Rocca et al. 2023).

Although these tools were developed roughly two decades ago, they have not been widely adopted in the study of social cognition in AUD. Instead, studies not employing classical tasks have primarily relied on self-reports to investigate related social cognitive processes such as empathy (Kumar et al. 2022). Beyond conceptual issues related to the definition and measure of empathy, it is important to stress that self-reports assess different constructs than behavioral tasks. Self-reports offer key insights on the way individuals perceive their social abilities, with relevance for real-life behaviors (e.g., low self-perceived social abilities can reduce the willingness to engage in social interactions, hence reinforcing social isolation). But they crucially depend on one’s awareness of potential difficulties, and are also affected by social desirability biases, arguing against their use as objective measures of social cognitive abilities (Pabst & Maurage, 2023). It is thus important to develop valid and ecological objective measures of social cognition. So far however, evidence from *one* study using the MASC shows that individuals with AUD present with worse performance at this task than healthy controls, indicating difficulties in inferring mental states in others (Maurage et al. 2016). More evidence using these standardized tools is needed.

The utility of these video-based tools can be further enhanced by combining them with physiological assessments. This combination offers mechanistic insights into social cognition measures, by identifying lower-level processes that impact complex social cognition. It also partly overcomes limitations associated with relying solely on multiple-choice questions and affective labels in performance measures. For example, efficient social decision-making is anchored in preserved perceptive and attentional processing of social stimuli, and training basic tangible perceptual/attentional processes can thus provide unique tools for treatment. To this end, a recent study by Patel et al. collected eye-gazing information during TASIT to inspect the role of visual scanning in social inference in individuals with schizophrenia. Using this integrative approach, the authors identified critical differences in visual scanning abilities in individuals with schizophrenia compared to controls, expanding on the evidence of reduced ability to detect sarcasm in this group (Bora and Pantelis 2016; Kern et al. 2009; Leitman et al. 2006). These results highlight the integrative role of visual perception in guiding the understanding of dynamic social scenes. Perhaps more importantly, it provides a biomarker of impaired social inference in schizophrenia. In severe AUD, there is emerging evidence suggesting variations in gaze patterns when individuals observe static facial expressions (Pabst et al. 2023; Pabst, in press 2023) and impairment of basic perceptive abilities in emotion decoding performance (Creupelandt et al. 2019). These recent studies represent a mechanistic advance in the approach to studying social cognition, since they involve the integration of traditional emotion recognition tasks with sensory perception measures. To enhance the validity of these findings, replication using more realistic stimuli, like those found in the MASC and TASIT, is important.

The use of video-based social stimuli described above can provide a rich characterization of social cognition, using stimuli with good ecological validity. While characterizing fine-grained perceptual mechanisms associated with altered social cognition is of critical importance, a precise assessment of aspects of social behavior can offer critical insights into the interactive aspects of socialization. Once more, the field of schizophrenia research provides pioneering methods in the quantification of social behavior in naturalistic settings. Birnbaum et al. developed a machine-learning based tool that allows to separate features of socialization uniquely based on facial and vocal digital information in an interactive setting (Birnbaum et al. 2022). The system integrates specific facial action units, such as for example cheek-raising, chin-raising and lip-pulling, and vocal features such as energy, frequency and spectral harmonicity, extracted using the OpenFace (Baltrušaitis 2016) and OpenSMILE (Eyben 2010) softwares. Based on digital data recorded during clinical interviews, the algorithm could classify individuals with schizophrenia from people with bipolar illness and healthy controls (Birnbaum et al. 2022). In addition, specific psychiatric symptoms such as affective flattening, apathy and anhedonia could be inferred from facial and acoustic features, alone or combined, a feature that can for example support clinical observation in objectively monitoring changes in negative symptoms. Finally, the use of both visual and auditory modalities contributed to discerning sex differences in the expression of schizophrenia versus bipolar disorders. Specifically, classification based on facial features was effective for men, while acoustic features proved valuable for women (Birnbaum et al. 2022). This finding shows that sex can be a critical determinant in the characterization of social processing in psychiatric conditions. It underscores the importance of incorporating sex as a factor when measuring social cognition in AUD.

Overall, this tool has the benefit of providing a standardized and objective assessment in a realistic clinical setting, allowing identification and monitoring of clinically meaningful impairments in social behaviour. Crucially, it could also offer the first insights into how individuals with AUD behave in social contexts, whereas previous studies have focused on the decoding of social signals generated by other individuals.

Social inference cognition hinges on efficient multi-modal perception and action. In this section, we highlighted the need for establishing a consensus on measures of social cognition in AUD, and presented approaches that can mitigate the long-standing trade-off between ecology and experimental control. The use of automated tools in the dissection of social interaction can provide outcomes that are more relatable to the observations used in animal research. In addition, combining measures of task performance with objective perceptual and behavioral profiles of social behavior can point to treatment strategies, by identifying more tangible biomarkers of social disfunction. In the next section, we argue for the importance of assessing social cognition along the clinical continuum, and highlight the importance of cross-species studies.

## Assessing the clinical continuum

Much like AUD is a chronic condition that develops gradually over time, the impairment of social function and marginalization unfold as a prolonged consequence of problematic drug use. In vulnerable individuals, alcohol initially tends to be consumed in a recreational manner, often in social settings. As alcohol use becomes more compulsive, it substantially impacts the individual’s ability to function socially. One of the contributing factors to the social impairments observed in individuals with AUD might be the neurological injury caused by alcohol. Meta-analytic findings show that individuals with AUD exhibit gray matter atrophy in regions involved in high-order executive functions, salience attribution and compulsivity (Xiao et al. 2015). This can have far-reaching implications that likely extend beyond alcohol-related behavior and should be considered as a potential contributor to social cognition disturbances.

Studies of patients in advanced stages of AUD are important for understanding the clinical condition. However, to understand how social impairments develop with progression of AUD, and to what extent they are consequences of alcohol problems or are pre-existing factors, studies of people who have not yet developed advanced AUD are essential. Understanding social cognition in individuals *at risk* of developing AUD, can contribute to the understanding of the interaction between alcohol use and impairments in social cognition. In a recent study of adolescents who engage in nonsuicidal self-injury (NSSI), we identified a relationship between self-harming behavior and perceived rejection (Perini et al. 2019). Using an interactive social task in the MRI scanner, we found that, compared to controls, adolescents with NSSI reported feeling more rejected, were more sensitive to rejection and disliked seeing their own face. Perceived rejection was associated with the frequency of self-harm, a finding that underscores the association between people’s subjective experience of social interactions and their engagement in harmful behaviors. While self-harm and harmful drinking are formally distinct behaviors, they often serve as means of experiential avoidance to cope with unwanted internal states, such as feeling rejected by others (Hayes et al. 1996; Kingston et al. 2010). Whether a negative social bias might be present at early stages of harmful drinking or might develop because of chronic problematic alcohol use is an empirical question that requires investigation.

Evidence from experimental cross-sectional designs at different stages on the clinical continuum can shed light on features that remain relatively stable or are malleable over time. For instance, in a recent study using two large cohorts, individuals with AUD had lower prosocial behavior than controls, shown by prototypical behavioral economic measures of altruistic behavior, fairness decision and reciprocal trust (Jangard et al. 2022). In agreement with these findings, in a preregistered randomized placebo-controlled study in a large sample of healthy social drinkers we showed that severity of alcohol use, assessed with AUDIT, was negatively associated with utilitarian and prosocial behaviors (Karlsson et al. 2022). Similarly, preliminary evidence suggests that drinkers with subclinical problems, such as young binge drinkers, have impaired social cognition abilities (Lannoy et al. 2021). Finally, two recent studies showed social cognitive impairments in offspring of people with AUD, who are at greater risk of disease, but are themselves symptom-free (Khemiri et al. 2022; Schmid et al. 2023). This consistency of findings across studies encompassing clinical and non-clinical populations demonstrates that even among individuals without a formal AUD diagnosis, drinking severity affects social functioning in a manner similar to what is seen in those with AUD.

While integrating cross-sectional evidence can be informative in the understanding of the dynamics of social cognition across different levels of clinical severity, employing longitudinal methods can allow for more conclusive identification of factors potentially contributing to social deficits. Although important for advancing the field, the longitudinal designs pose significant challenges in humans. Furthermore, absent experimental interventions, human studies cannot probe the causal nature of associations between social factors and alcohol use severity. Fostering a close translational dialogue could mitigate these challenges, provide complementary insights, and substantially improve our understanding of social cognition in AUD. Rodent models may offer unique opportunities for understanding the intricate interactions between social behavior and alcohol-related behaviors, making it possible to address research questions that are challenging or in some cases, not feasible to address in humans. Rodent models can exploit individual differences in heterogeneous populations, and the relationship between social behaviors and, for instance, excessive choice of alcohol over natural rewards (Augier et al. 2018), or self-administration despite negative consequences, often referred to as compulsive use (Domi et al. 2021). Once established, these behaviors can be studied and manipulated over-time, allowing for longitudinal mechanistic inferences in a more time-efficient way than in humans. Pioneering preclinical work by Venniro et al. 2018; showed the feasibility of addressing social behavior in the study of addiction using a setup that balances ecology and experimental control, a trade-off often harder to obtain in human settings (Venniro et al. 2018). Their setup allows the animals to interact freely while monitoring behavioral features in an objective and standardized manner. In line with this, innovative work has measured the links between alcohol related behaviors and reduced sociability in rodents (Leclercq et al. 2020). We believe that human research in AUD can benefit from preclinical models that include social factors. However, the ability of preclinical research on social factors to inform human studies relies on a dialog between scientists studying animal models and humans. It is also important to consider that substantial species differences in social behaviors exist. For example, there has been an “evolutionary switch” from nocturnal to diurnal activity in primates (Shultz et al. 2011). Some differences across different rodent species should also be considered, such as variations in typical social dynamics in rats versus mice. For example, a recent study found substantial differences in social behavior across two mouse and rat strains (C57BL/6 J and Sprague Dawley respectively), with the rat strain being less aggressive and more sociable that mouse strain (Netser et al. 2020). It is therefore important to consider what species that is optimal to use in models of social behaviors, and what measures that are likely to provide insights that translate across species.

Social behavior is malleable over time. The social difficulties observed in individuals with AUD might manifest differently at earlier stages of problematic alcohol use. In this section we highlighted the importance of addressing the temporal emergence of social impairments in AUD, with improved prevention strategies being one potential benefit. To this end, human research can benefit from animal models, and the integration of cross-species evidence can help moving the field forward. Identifying consistent traces and distinguishing features of social dysfunction across varying severity levels can help in preventing and treating AUD. The next section focuses on the importance of factors that are stable over time, namely personality traits.

## A role for personality traits and sex

Examining social functioning across different severity levels can help evaluate factors that might change over time, whereas considering personality traits and sex can offer insights into the contribution of relatively stable factors. This section does not intend to provide an extensive review on these topics but highlights the importance of including personality traits and sex for a more nuanced understanding of the complex interplay of variables influencing social cognition.

Personality traits have been studied in association with alcohol use, a literature recently integrated a meta-analysis (Hakulinen et al. 2015). The authors used an individual-participant meta-analytical approach to examine how personality traits, based on the the Five-Factor Model, relate to self-reported alcohol use. This meta-analysis included both cross-sectional and longitudinal studies. Both approaches revealed that higher levels of extraversion and lower levels of conscientiousness were associated with an increased likelihood of transitioning from moderate to heavy alcohol use. Individuals with lower conscientiousness tend to be impulsive, and there is extensive evidence for an association between impulsivity and drug use and abuse (de Wit 2009). Consistently across task-based and self-report measures, individuals with SUD including AUD show higher impulsivity than controls (de Wit 2009; Verdejo-Garcia and Albein-Urios 2021). Furthermore, longitudinal research summarized in a recent review (Verdejo-Garcia and Albein-Urios 2021), indicates that impulsive adolescents and young adults have an increased risk of developing SUD later in life. Conversely, according to the meta-analysis mentioned above (Hakulinen et al. 2015), lower levels of extraversion and openness, coupled with higher levels of agreeableness, showed a protective effect, and were related to higher likelihood for transitioning from moderate alcohol intake to abstinence. Results from our behavioral economics study show an interesting pattern: while alcohol use severity was associated to decreased utilitarian and prosocial behaviors, agreeableness had instead a positive association (Karlsson et al. 2022). This result suggests that one of the reasons high agreeableness may serve as a protective trait against AUD (Rawls et al. 2021) might be because of its potential role in increasing prosocial behavior.

Another central “behavioral trait” in the characterization of AUD is antisocial behavior. Antisocial traits in childhood have long been associated with early onset of alcohol use and increased risk of developing AUD later in life (Cadoret et al. 1995; Clark et al. 1998). In adults, antisocial personality disorder (ASPD) has a negative association with conscientiousness and agreeableness (Saulsman and Page 2004, 2005). Meta-analytic evidence shows that these traits are central to the AUD profile (Samuel and Widiger 2008), and predict drinking tendencies over time (Hakulinen et al. 2015). The consistent overlap in personality traits between ASPD and AUD is further highlighted by their high co-occurrence rates (Helle et al. 2019), and overlapping genetic architecture (Kendler et al. 2003). However, AUD is highly heterogeneous (Litten et al. 2015). An example of this heterogeneity comes from the seminal classification by Cloninger and colleagues (Cloninger et al. 1981). Clinical presentation that is the most common in men is indeed characterized by early onset and impulsive and aggressive tendencies, resembling traits found in ASPD (Cloninger 1987). Cloninger and Bohman also described a different clinical profile, characterized by late onset and anxious rather than impulsive traits. While different AUD subtypes have been described over the years (Leggio et al. 2009), these two profiles highlight the heterogeneity of AUD, and may reflect fundamentally different disease mechanisms, rooted in internalizing versus externalizing processes. Consistently, personality traits were also proven informative in supporting the three-factor model of addiction (Koob and Volkow 2010; Kwako et al. 2016) for alcohol use disorder (Kwako et al. 2019), with neuroticism, extraversion, agreeableness and impulsivity having significant loadings to the “negative emotionality” and “executive function” factors, (Kwako et al. 2019). Overall, including personality traits alongside measures of social cognition may be helpful in two ways. First it would help in understanding their influence on social cognition in individuals with AUD, or at risk of developing AUD. It will also help to disentangle factors that alter social cognition but are independent, and likely preceding problematic substance use.

As with personality traits, sex is also a factor that can provide insights in understanding social cognition in AUD. Currently, there is a need for further research regarding sex differences in social cognition in AUD, as the existing evidence does not provide a cohesive picture. As mentioned in the section above, meta-analytical findings are mostly inconclusive. The most recent meta-analyses available on emotion recognition (Bora and Zorlu 2017; Castellano et al. 2015) and theory of mind (Onuoha et al. 2016), include studies with low sample size and often largely unbalanced representation of sexes in the samples, precluding systematic statistical analyses. Hence, it is currently unclear whether, and for which specific processes or severity levels, sex might affect social cognition in AUD. Addressing these questions is essential from a fundamental and precision-medicine perspective. We therefore encourage future studies to consider larger sample sizes, with a particular emphasis on achieving a better balance in the representation of sexes to allow for a more comprehensive investigation.

## Conclusion

Social cognition should be regarded as a defining component of AUD. This brief commentary underscores the need to dissect social cognition and behavior using available tools that allow standardized measures using real-world settings. In addition, it underscores the importance of recognizing the malleable nature of social processes over time by assessing social cognition in individuals who are at risk of developing AUD. This assessment should aim to distinguish between stable, trait-like aspects of social cognition, and those that change over time, thereby enhancing opportunities for prevention. With the same goal, and given their critical role in defining problematic substance use, personality traits need to be incorporated in the investigation of social cognition, to uncover their potential contribution to the social disturbances observed in AUD.
